# Development and Evaluation of a Polyvinylalcohol -Cellulose Derivative-Based Film with Povidone-Iodine Predicted for Wound Treatment

**DOI:** 10.3390/polym12061271

**Published:** 2020-06-02

**Authors:** Dorota Kida, Olimpia Gładysz, Małgorzata Szulc, Jacek Zborowski, Adam Junka, Maciej Janeczek, Anna Lipińska, Aleksandra Skalec, Bożena Karolewicz

**Affiliations:** 1 Department of Drug Form Technology, Wroclaw Medical University, Borowska 211 A, 50-556 Wroclaw, Poland; dorota.kida@umed.wroc.pl; 2 Department of Analytical Chemistry, Wroclaw Medical University, Borowska 211 A, 50-556 Wroclaw, Poland; olimpia.gladysz@umed.wroc.pl; 3 Department of Periodontology, Wroclaw Medical University, Krakowska 26, 50-425 Wroclaw, Poland; malgorzata.szulc@umed.wroc.pl (M.S.); jacek.zborowski@umed.wroc.pl (J.Z.); 4 Department of Pharmaceutical Microbiology and Parasitology, Wroclaw Medical University, Borowska 211 A, 50-556 Wroclaw, Poland; adam.junka@umed.wroc.pl; 5 Department of Animal Physiology and Biostructure, Wroclaw University of Environmental and Life Sciences, Norwida 25, 50-375 Wroclaw, Poland; maciej.janeczek@upwr.edu.pl (M.J.); anna.lipinska@upwr.edu.pl (A.L.); aleksandra.skalec@upwr.edu.pl (A.S.)

**Keywords:** povidone-iodine, polymer film, cellulose derivatives, polyvinyl alcohol, rat model, tissue regeneration

## Abstract

The aim of this study was to develop and assess a polyvinyl alcohol-cellulose derivatives-based film with incorporated povidone-iodine (PVP-I) predicted for applications in the treatment of periodontitis. Films were fabricated by solvent-casting, and their physical characteristics, such as their surface and structure morphology, mechanical properties, and disintegrating time, were evaluated. For in vitro iodine release studies and evaluation, the antimicrobial activity was tested using a modified disc diffusion method against five microbial strains. For further use, we selected the film with polyvinyl alcohol-hydroxypropyl methylcellulose (PVA/HPMC_B) based on acceptable physicochemical properties. To assess the subacute toxicity of the film composition, the tissue regeneration process was tested in rats and compared to a conventional dressing commonly used in wound healing (Spongostan). Seven days after implantation, dorsal skin sections and blood samples (n = 10, in total n = 30) were examined. The wound area, epithelium, and dermis were evaluated microscopically, while the blood collected from the rats underwent biochemical analysis. The blood biochemistry results were comparable in all three groups. No significant histological differences between the Spongostan and the placebo film developed after subcutaneous implantation were observed. In contrast, the inflammation stage was reduced and the “scar” in the dermis was smaller when PVP-I and PVA/HPMC_B films were used. A smaller local inflammatory response inflicted less tissue damage, leading to the activation of subsequent regeneration phases and restoration of the area to its original state. The results obtained confirmed that PVP-I incorporated into PVA-hydroxypropyl methylcellulose film is a promising drug carrier, working faster and more effectively than the other two dressing materials evaluated. These developments provide a promising alternative in tissue regeneration and the wound healing process.

## 1. Introduction

The skin forms the outer layer of the integumentary system, and its thickness varies in different areas of the body. Skin is formed from bundles of collagen and elastic fibers suspended in a small amount of acellular basal material that contains inactive fibroblasts. When activated, these fibroblasts produce collagen, elastin, and the intercellular matrix. The skin is divided into three layers: the epidermis, dermis, and subcutaneous tissue.

The epidermis is the dynamic, most external layer. The dermis (*cutis vera*) is located immediately under the epidermis and consists of a papillary layer and a reticular layer. It possesses collagen and elastic fibers, skin receptors, blood vessels, nerves, sweat glands, and sebaceous glands, as well as hair follicles. The subcutaneous tissue is the last and deepest layer (*hypodermis, tela subcutanea*) and consists of loose connective tissue with fat cells, lower hair roots, and glandular secretory units, as well as some receptors [1,2].

Wounds form as a result of damage that disrupts the integrity of the skin. Extensive and complex wounds, as well as those that do not easily heal, may lead to emaciation or even death. Wounds are divided into acute or chronic [3], superficial, intermediate, and deep [4]. However, regardless of the type and depth of damage, the wound healing process includes mutually independent, successive phases [5,6].

The aim of the healing process is to induce hemostasis and transition into post-inflammatory phases. If the inflammatory phase is prolonged, the subsequent phases also undergo elongation, and the healing process is delayed [7]. Two mechanisms play a key role in the healing process. The first involves a contraction of the wound and epithelium, and the second leads to the formation of new skin tissue associated with epithelial growth, which also acts to prevent wound contraction [8]. In mice, wounds mainly close in response to the contraction mechanism, while in humans, the process largely takes place due to epithelial growth. Wound contraction may cause scar contracture, limiting skin mobility and sometimes causing scar hypertrophy. The scar, which is a zone of dysfunctional and structurally isolated tissue, is formed from fibrous connective tissue and constitutes a stage of remodeling. It may be moderated by shortening the healing time and reducing tissue damage, which can be regulated by reducing the inflammatory reaction [9].

Therefore, the selection of an appropriate and safe hemostatic dressing that does not induce pathological reactions is of the utmost importance in wound healing. Despite the availability of various substances that optimize the wound healing environment, the search for new and more effective products continues. In the past, dressing materials aimed to dry the wound, limit exudates, and inhibit bacterial growth [10]. However, later research indicated that a moist and warm environment is more suitable for wound healing [4]. Such conditions enable the migration of epithelial cells to the site of damage [11]. In a moist wound environment, the number of endothelial cells and fibroblasts increases, while inflammatory cells (neutrophils and macrophages) reproduce more slowly [12]. Moreover, moist wounds are less prone to scab formation, which minimizes both cosmetic concerns [13,14] and pain [15]. There are various dressing types, such as polymer dressings (foams, hydrogels, alginates, and hydrocolloids) or natural films and skin substitutes which contain live cells. However, there is no all-purpose dressing for all wound types. Existing polymer dressing types, such as polymer foams, hydrogels, alginates, hydrocolloids or natural films, and skin substitutes which contain live cells, have their own advantages; however, they do not have antimicrobial properties. In our research, we have worked to develop a polyvinyl alcohol (PVA)-cellulose derivative-based film with incorporated povidone-iodine. Ultimately, this film can be used to treat periodontitis and its influence on the local response and process of tissue regeneration were referred to commercial dressing Spongostan, commonly used in the healing of dental wounds.

Spongostan is a passive dressing available in the form of a sponge intended to stop bleeding in areas of high humidity and those that are difficult to access. Due to the use of pork or fish gelatin, it is a soluble hemostatic product [16] that is capable of limiting focal bleeding, while remaining completely resorbable in areas of tooth extraction or bone grafts [17]. The total resorption time of Spongostan may reach six weeks [18] without inducing pathological tissue reactions, and at the same time, maintains patient safety.

PVP-I (iodinated polyvinylpyrrolidone, classified as an iodophor) is a water-soluble complex of iodine with a povidone polymer that releases iodine for some time following administration. Therefore, it provides a strong antibacterial profile against Gram-positive and Gram-negative microorganisms. It is less irritating than other aqueous solutions of iodine, and is less active, but also less toxic, than free iodine. After contact with skin or mucous membranes, PVP-I penetrates deep into the tissues and undergoes slow degradation, during which time the polymer releases increasing amounts of iodine. The rest of the substance diffuses back to the skin surface [19]. Iodine destroys the amino acids that form the enzymes and structural proteins of microorganisms, leading to protein inactivation or damage. Such destructive activity persists for some time, providing the substance with a prolonged effect. The amount of substance absorbed through the skin is inversely proportional to the skin’s thickness; that is, the thinner the skin, the more substance that is absorbed, e.g., in a newborn [20]. PVP-I is used prophylactically to treat skin and mucous membrane infections, acute and chronic fungal or parasitic infections (candidiasis and trichomoniasis), and non-specific or mixed infections, as well as to disinfect the skin. In stomatology, it is used for subgingival irrigations, scaling [21,22], to reduce the periodontal pocket depth, to improve the connective tissue attachment in deep periodontal pockets, and to reduce the number of pathogenic microorganisms in the periodontium [23,24]. In addition to dentistry, PVP-I is widely and successfully used in surgery, dermatology, pediatrics, urology, and gynecology.

The aim of this study was to develop a PVA-cellulose derivative-based film with incorporated PVP-I. We went on to assess the subacute toxicity after subcutaneous implantation of the film and compared its effectiveness with that of commercial dressing in tissue regeneration in a rat model. In addition, we sought to establish whether the healing process would be shortened with the use of the film with PVP-I, as well as identify the histological tissue changes occurring during this process.

## 2. Materials and Methods

### 2.1. Film Preparation

Film formulations were prepared using the solvent-casting technique [25]. Dressings based only on PVA polymer possess inadequate mechanical properties which restrict their use as wound dressings. The proposed film combines PVA and chosen swelling and gelling biocompatible polysaccharides allow the mechanical properties and disintegration process of solid membranes to be adjusted to requirements for wound healing materials. The composition of fabricated films is listed in Table 1. The calculated amount of hydroxypropyl methylcellulose (HPMC) (40–60 mPa·s, Sigma, St. Louis, MO, USA) or carboxymethylcellulose sodium salt (CMC) (50–200 mPa·s, Sigma, St. Louis, MO, USA) was added gradually while stirring (L366, Labinco BV, Breda, The Netherlands) to half the required volume of cooled distilled water. Then, the PVP-I (Sigma, St. Louis, MO, USA) solution was added. Polyvinyl alcohol (PVA) (24–32 mPa·s, Avantor Performance Materials Poland S.A., Gliwice, Poland) was dissolved in hot water, added to obtained cellulose derivative-PVP-I gel, and homogenized for 1 h (h), adapting the rotation speed to the viscosity of the dispersions. Finally, the specified volume of glycerol (GLY) (Sigma, Steinheim, Germany) was added to the mixture during stirring. The mixture was sonicated to remove air bubbles until a clear solution was obtained. Next, 35 g of this dispersion was slowly poured into a Teflon-coated Petri dish and dried in an air circulating oven at 40 °C for 24 h, resulting in a thin film after solvent evaporation. The features of the product were evaluated, including the homogeneity, smoothness, presence of bubbles, and ease of separation from the dish. The dried films were divided into 10 × 25 mm inserts, and samples were stored in the dark in a sealed desiccator until further analyses.

### 2.2. Characterization of the Dried Films

The fabricated films were evaluated for thickness and mass uniformity. The thickness of the films at six spots (two at the center and four at the different corners of the film) on an original uncut film was measured using a digital micrometer screw gauge (Absolute AOS ABS, Mitutoyo, Novi, MI, USA), and the mean value was reported. For mass uniformity, cut samples (10 × 25 mm) were weighed individually using an electronic balance (Pioneer, Ohaus Corp., Parsippany, NJ, USA) and recorded as the mean of six measurements.

#### 2.2.1. Surface Morphology Observation

The characterization of the surface morphology of the optimized dried films was carried out using a scanning electron microscope (SEM Auriga60 Zeiss, Jena, Germany, ion sputtering EM ACE600, Leica, Germany) with a spatial resolution of 3 nm at 30 kV. The samples of the cut optimized film were separately fixed to metal stubs, which were then gold-sputtered using ion sputtering under vacuum. After gold coating, scanning electron microscopy was used to obtain images of the samples at a magnification of 10,000×.

#### 2.2.2. Mechanical Property Testing

The mechanical properties of the optimized film were evaluated using a TA-XT plus texture analyzer (Stable Micro System, Godalming, UK) in a simple tension mode at ambient temperature. Samples of the films were held by two clamps (tensile grips Type A/TG) at an initial separation distance of 2 mm at a constant speed of 1 mm s^−1^.

#### 2.2.3. Disintegrating Time

The film disintegration was represented as the time required for the film to undergo full dissolution. Samples with a size of 10 × 25 mm were immersed in 5 mL PBS and incubated at 37 °C in a sealed closed vessel with horizontal shaking 60 times per minute. The test was continued until the original film entirely dissolved in the medium. The test was repeated six-fold for each formulation.

#### 2.2.4. pH Film Studies

The pH of the film was determined in order to investigate the possible side effects due to a change of pH in vivo, since an acidic or alkaline pH may cause tissue irritation after implantation [26]. The film samples (10 × 25 mm) were placed in a flask with distilled water (3 mL) and mixed to obtain an aqueous extract. Next, a pH meter electrode was immersed into the extract and allowed to equilibrate for 1 min, and the pH was noted. The mean of six determinations was recorded.

### 2.3. Fourier Transform Infrared (FTIR) Spectroscopy

The excipient–drug interactions in the film were examined at room temperature using a Fourier Transform Infrared (FTIR) spectrophotometer (Thermo Nicolet iS50, Waltham, MA, USA). The FTIR values of the pure drug, excipients, and optimized film with PVP-I incorporated were noted in the frequency range of 500–4000 cm^−1^ at a resolution of 4 cm^−1^. The scan speed was 0.2 cm/s.

### 2.4. In Vitro Iodine Release Studies

#### 2.4.1. Determination of the Iodine Content in Povidone-Iodine

Iodine (I_2_) determination in PVP-I was based on the titrimetric method recommended by Polish Pharmacopeia XI [27]. A total of 1.0 g PVP-I was dissolved in 150 mL demineralized water in an Erlenmeyer flask with a cap and mixed on a platform shaker for 1 h in darkness. After the addition of 0.1 mL acetic acid (115–125 g·dm^−3^), the solution was titrated using a solution of sodium thiosulfate (0.1 moL dm^−3^ solution, made of Na_2_S_2_O_3_ 5H_2_O, FIXANAL) in the presence of starch as an indicator. Starch solution (1%) was prepared by the dissolution of triturated starch in boiling water. It was used fresh and at ambient temperature. In these experimental conditions, iodine was reduced by thiosulphate according to Equation (1):

(1) $$I_{2\;}+2S_{2}O_{3}^{2-}\to 2I^{-}+S_{4}O_{6}^{2-}.$$

The determined available content of 11.4% I_2_ in PVP-I was in agreement with the manufacturer’s declaration (9–12% I_2_) and Pharmacopoeia requirements stated in the monograph [27].

#### 2.4.2. In Vitro Iodine Release Studies

Three samples of the film incorporated with PVP-I were immersed in 50 mL water and mixed using a magnetic stirrer at 37 °C at 250 rpm. In order to protect them from light, the samples were stirred in orange flasks with screw caps and wrapped in aluminum foil. The determination of I_2_ during release studies could not be carried out by titration due to the relatively small amount of I_2_ in the acceptor medium. An alternative to I_2_ determination is UV-VIS spectroscopy [28], which also avoids irreversible usage of the iodine solution. Samples of the 3.5 mL solution were taken at 20-min intervals for 2 h, spectrophotometrically measured, and returned to the stirred solution. The experimental conditions remained in a “sink” state due to the good solubility of PVP-I in water. The Cs > 10 Ct requirement (where Cs is the concentration of the saturated solution and Ct is the concentration after t time of release) was also met [29]. When PVP-I was dissolved in water in the presence of I_2_, according to Equations (2) and (3) (below), I_3_^−^, I^−^, and HIO species were also formed [28]:

(2) $$I_{2}+H_{2}O\;\to \mathrm{HIO}+I^{-}+H^{+},$$

(3) $$I_{2}+{\;I}^{-}\to I_{3}{}^{-}.$$

**Figure 1:**
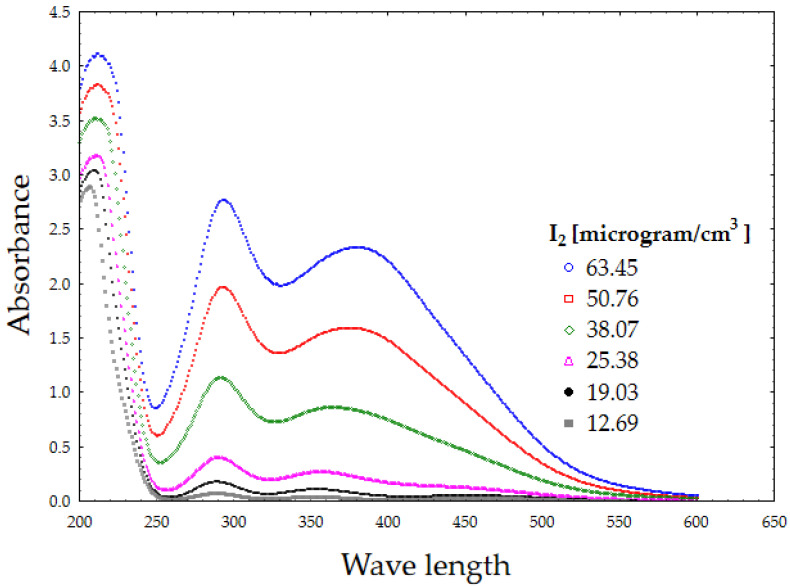
UV-VIS spectra of povidone-iodine (PVP-I) standard solutions.

The presence of two clear and distinctive peaks, attributed to I_3_^−^ (at 288 and 360 nm), was used for PVP-I content determination. The peak at 225 nm was related to I^−^ presence [28,30]. In order to calculate the calibration curve for spectrophotometric measurements, standard solutions were prepared by dissolving PVP-I and diluting it to an iodine concentration of 63.45–12.69 µg cm^−3^. Their UV-VIS spectra were recorded (Figure 1). Based on the absorbance measurements, two calibration curves were calculated for the wavelengths of 288 and 360 nm using Equations (4) and (5), with R^2^ estimated as 0.98.

(4) $$A_{288\;\mathrm{nm}}=0.0535{\;C}_{I_{2}}\left[{\mu g\;\mathrm{cm}}^{-3}\right]-0.8019$$

(5) $$A_{360\;\mathrm{nm}}=0.0452{\;C}_{I_{2}}\left[{\mu g\;\mathrm{cm}}^{-3}\right]-0.7258$$

### 2.5. Estimation of the Antimicrobial Activity of the Selected Polyvinyl Alcohol-Hydroxypropyl Methylcellulose Film (PVA/HPMC_B) with Incorporated PVP-I Using a Modified Disc Diffusion Method

The following strains from the American Tissue and Cell Culture Collection (ATCC) were applied: *Staphylococcus aureus* 6538; *Pseudomonas aeruginosa* 15442; and *Enterococcus faecalis* 29212, *Candida albicans* 10231. Additionally, *Lactobacillus rhamnosus* strain 489 from the Polish Microbiology Collection (PCM) was applied. The sterile films of PVA/HPMC_B with incorporated PVP-I were placed on the surface of the M-H agar medium (BioMaxima, Poland) seeded with the suspension of *S*. *aureus*, *P. aeruginosa*, *E. faecalis*, and *C*. *albicans* at a density of 0.5 McFarland. In the case of *L. rhamnosus,* Rogosa Agar was applied. Next, 40 µL of sterile water was poured on the dressings to unfold them. Subsequently, the cultures were left at 37 °C for 24 h. The results are presented as the diameter of the growth inhibition zone (mm) minus the dressing diameter (mm). The tests were performed in triplicate.

### 2.6. Assessment of Polymer Material after Subcutaneous Implantation in Rats

#### 2.6.1. Animals and Experimental Design

The study included 30 female rats weighing approximately 250 g. Following a seven-day acclimatization period, the rats were divided into three groups (one control group and two study groups). Each included 10 animals. The animals were deeply anesthetized following an intramuscular administration of medetomidine (90 mg/kg bw) and ketamine (0.5 mg/kg bw) and underwent the surgical procedure, which was performed in the rib area. An area of shaved skin in the lateral rib area was incised. Subsequently, a subcutaneous pocket was formed, where one of the studied films was administered in the form of a 5 × 5 mm square (Spongostan, Ferrosan Medical Devices, Søborg, Denmark placebo film PVA/HPMC_B without the drug, or PVA/HPMC_B film with incorporated PVP-I). After implantation, the wound was sutured with Amifil 5-0^®^ suture material, and buprenorphine (0.1 mg/kg bw) was subcutaneously administered. No decrease in food or water intake was observed during the observation period, and the behavior of the rats appeared normal. After seven days, the rats were anesthetized once again, according to the above-described protocol. Skin sections were collected from the implantation areas. The 5 × 5 mm sections underwent standard procedures to yield histological samples. The skin was sutured using the Amifil^®^ 5-0 suture material. Blood samples from the tail vein were collected when the animals were anesthetized the second time. In each group, the blood biochemistry and acute-phase protein C-reactive protein (CRP) were measured in five animals, while the complete blood count was recorded in five other animals. Blood analysis was divided in this way because the amount of blood collected from one animal was insufficient to perform all analyses. The blood was collected from the tail vein because, in accordance with the recommendation of the bioethics committee, the animals were put up for adoption following the study. The research was approved by the Local Bioethics Committee No. 021/2019/P1.

#### 2.6.2. Histopathological Analysis

All samples were fixed in 4% buffered formalin and embedded in paraffin blocks. The 5 µm paraffin sections were obtained using a rotary Hyrax M25 (Carl Zeiss, Jena, Germany microtome. They were then stained with hematoxylin and eosin (HE), as well as Masson trichrome (MTC), according to appropriate histological protocols. The analysis of the skin cross-sections was performed using an optical microscope (Axio Scope^®^ A1; Carl Zeiss).

#### 2.6.3. Blood Biochemical Analysis

Blood biochemical analysis was performed using the CatalystOne^®^ (Idexx, Westbrook, ME, USA) system. The blood samples for haematological and biochemical analysis were prepared according to standard protocols of the ProCyte Dx Haematology Analyser for blood cells and the Catalyst One (Idexx) System for biochemical analysis.

## 3. Results

### 3.1. Physicochemical Characteristics of the Polymer Film

PVA has excellent and straightforward film-forming properties and has been blended with cellulose derivatives to improve the mechanical and physicochemical properties of developed polymeric films [31,32]. The final properties of the polymer material depend on the properties of the embedded components, including added plasticizers, so the PVA film properties change after blending.

The products obtained from both cellulose derivatives were yellow, transparent, and characterized by different physical properties according to the two types of cellulose derivative. The higher applied viscosity of the carboxymethylcellulose sodium salt in formulation resulted in the collapse and roughness of the obtained film surface. Increasing the concentrations of both cellulose derivatives in film formulations resulted in them becoming soft and even sticky, leading to difficulties in separating them from the dish. Therefore, higher concentrations were excluded from the study. In the case of the lower viscosity cellulose derivative used at the same time as a higher concentration of PVA (PVA/HPMC_B), the film was homogeneous, with a smooth surface, and was durable, flexible, and easily pulled. The physical characteristics of the PVA/HPMC_B formulation selected for the in vitro drug release test, biological tests, and in vivo application are presented in Table 2.

**Figure 2:**
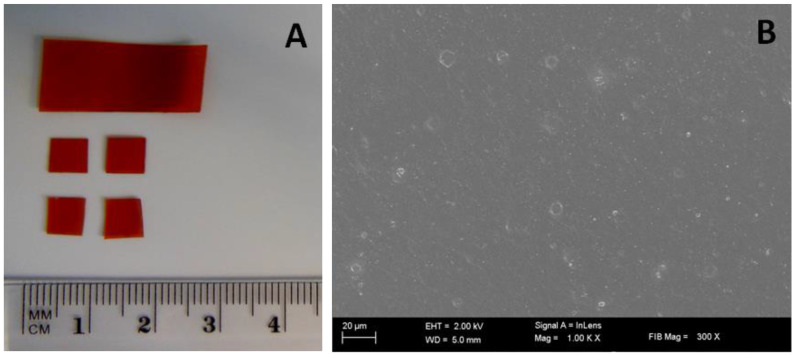
PVA/HPMC_B film samples with incorporated PVP-I. (**A**) An optical image of samples before assessment (picture taken with a Nikon S9300 camera using a macro lens); (**B**) representative scanning electron microscopy (SEM) image. Scale bar 20 µm (10,000×).

The selected film was easy to cut (Figure 2A), and high magnification imaging performed using SEM revealed a high smoothness and uniform surface, with no obvious porous regions of prototypical film with incorporated PVP-I. The small white circles seen in the SEM image are only due to the formation of small bubbles during drying (Figure 2B). The force required to elongate the film during the pulling process extended to a maximum of 5 kg applied by the analyzer. All film samples disintegrated within 120 min, and the pH of the extract after film disintegration ranged from 5.9 to 6.7.

**Figure 3:**
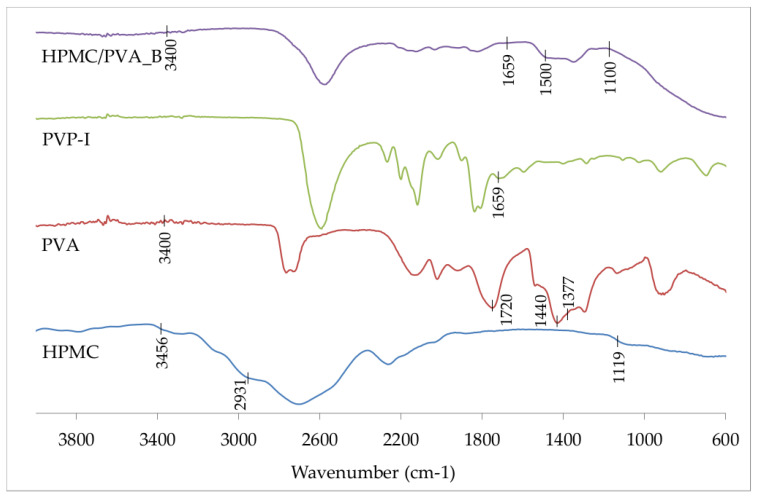
Fourier Transform Infrared (FTIR) spectra of pure hydroxypropyl methylcellulose (HPMC), pure PVA, PVP-I, and PVA/HPMC_B film with incorporated PVP-I.

Figure 3 shows the FTIR spectra of pure HPMC, pure PVA, PVP-I, and PVA/HPMC_B film with incorporated PVP-I. In particular, the FTIR spectrum of PVA reveals a broader peak of around 3400 cm^−1^ for the stretching vibration of -OH moieties due to the intra- and extra-molecular hydrogen bonding. Additionally, the peaks at 1720–1737, 1440, and 1377 cm^−1^ correspond to the stretching vibration of -C = O and bending vibrations of -CH_2_ and -CH groups, respectively [33]. In the FTIR spectrum of HPMC, the broad absorption band at 3456 cm^−1^ indicates the stretching frequency of the -OH groups, with peaks at 2931 and 1119 cm^−1^ signifying C-H stretch and C-O-C asymmetric stretch vibrations, respectively. The peak observed at 1659 cm^−1^ corresponds to the typical absorption of the carbonyl group in the pyrrolidone ring of PVP-I. In the FTIR spectrum of PVA/HPMC_B film with incorporated PVP-I, a decreased intensity was observed for the region 1100–1500 cm^−1^. Simultaneously, we found an absence of additional bands in the spectrum, except for those characteristic of formulation components at around 3400 cm^−1,^ indicating the presence of PVA and HPMC, and at 1659 cm^−1^, indicating the presence of PVP-I.

### 3.2. In Vitro Release of Iodine from the Film with Incorporated PVP-I

**Figure 4:**
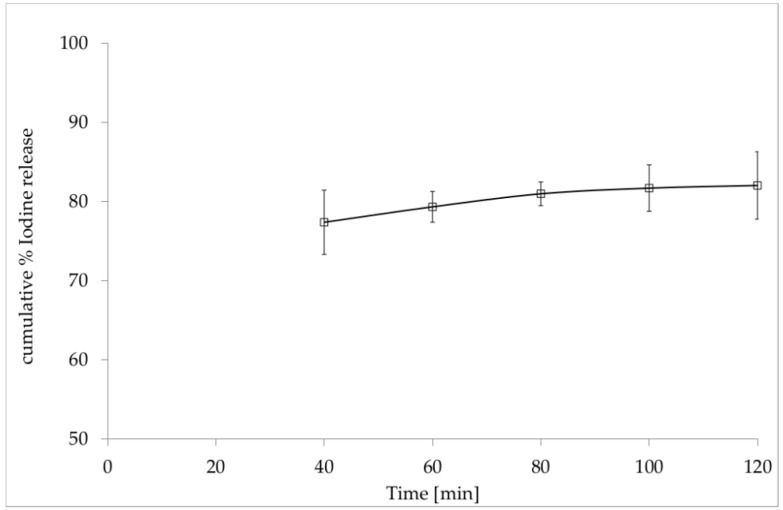
In vitro release profile of iodine (I_2_) from PVA/HPMC_B film (with incorporated PVP-I) versus time.

The UV-VIS spectra recorded for PVP-I solutions were compared to those of the film without PVP-I after total dissolution under the same conditions. In the range of 250–400 nm, we detected no significant absorption differences of the film ingredients. Figure 4 illustrates the changes in the molar iodine concentration as a function of time, i.e., the iodine release profile. The cumulative values of the percentage of iodine release (% I_2_ release) from PVA/HPMC_B film with incorporated PVP-I over time were calculated as shown in Equation (6):

(6) $$\mathrm{Cumulative}\;\left[\%\right]{\;I}_{2}\mathrm{release}=\frac{C_{I_{2}}\;\mathrm{released}\;}{C_{I_{2}}\;\mathrm{declared}}\times 100\%,$$

where $C_{I_{2}}$ released is the molar concentration of I_2_ calculated from the UV-VIS spectrum for 288 and 360 nm. $C_{I_{2}}$ declared is the molar concentration of the total I_2_ amount in the original sample (theoretically calculated based on the sample weight and its formulation). The cumulative percentage of release was calculated based on Equation (7):

(7) $$\mathrm{Cumulative}\;\left[\%\right]{\;I}_{2}\mathrm{release}\;=\frac{\mathrm{weight}\;\mathrm{of}\;\mathrm{drug}\;\mathrm{released}\;\left[\mathrm{mg}\right]}{\mathrm{weight}\;\mathrm{of}\;\mathrm{drug}\;\mathrm{in}\;\mathrm{the}\;\mathrm{sample}\;\left[\mathrm{mg}\right]}\times 100\%.$$

Figure 4 presents the average percentage of iodine release (average % I_2_ release) in the experimental time calculated from equations describing the release.

**Figure 5:**
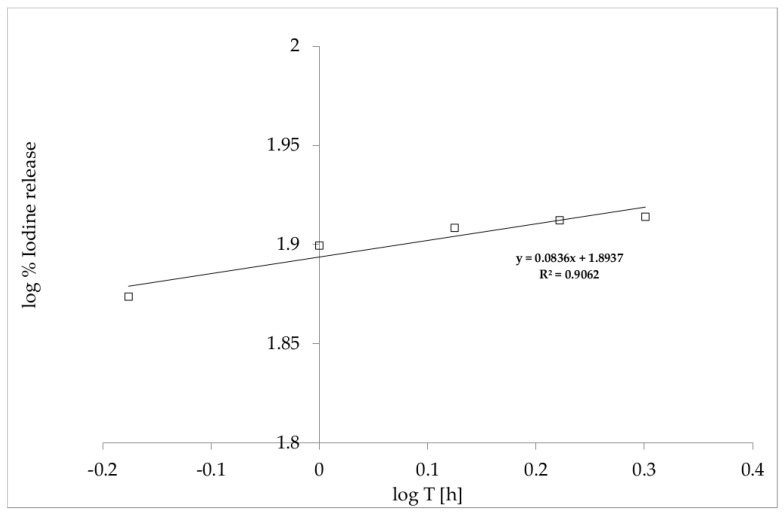
Korsmeyer–Peppas model of the kinetic release of I_2_ from PVA/HPMC_B film with incorporated PVP-I.

The findings displayed in Figure 4 show a non-linear-dependent I_2_ release profile for the film over time. Therefore, to interpret the release of the drug, the Korsmeyer–Peppas model presented in Equation (8) was used:

(8) $$\log\%\;\left(\frac{M_{t}}{M_{\infty }}\right)=n\;\log\;t\;+{\;\log\;K}_{\mathrm{kp}},$$

where log % ($\frac{M_{t}}{M_{\infty }}$) is the logarithm of % drug release, n is a diffusional exponent, and K_kp_ is the Korsmeyer release rate constant (Figure 5) [29,34]. The n value in the Korsmeyer–Peppas model indicates the mechanisms that should be used to describe how active compounds are released from their matrix. In this case, for n < 0.5, the solvent diffusion is much greater than the process of polymeric chain relaxation, and the kinetics of drug release from the film can be characterized as being dominated by a diffusion mechanism.

### 3.3. Evaluation of the Antimicrobial Activity of the Selected Film with Incorporated PVP-I Using a Modified Disc Diffusion Method

**Figure 6:**
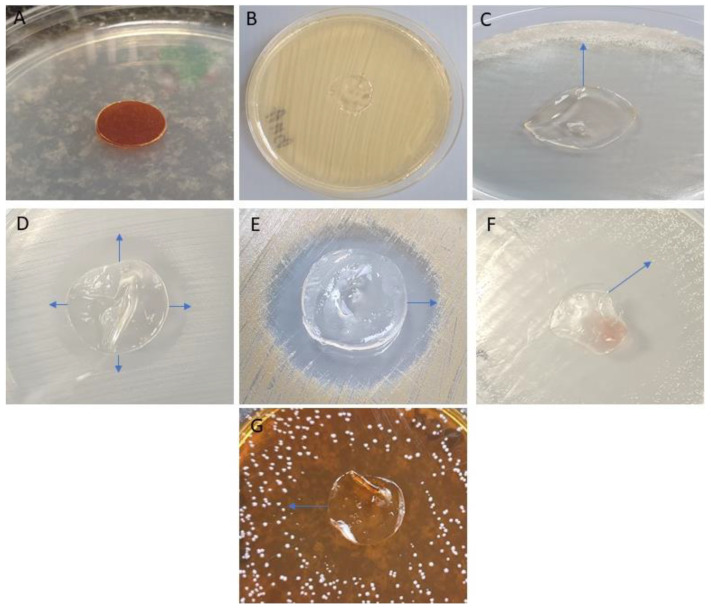
Antimicrobial activity of PVA/HPMC_B film with incorporated PVP-I. (**A**) The film placed on the sterile agar plate surface; (**B**) control representative setting: film without PVP-I placed on the *S. aureus* lawn displays no antimicrobial activity (no inhibition zone is observed); (**C**) inhibition zone of *C. albicans* growth; (**D**) inhibition zone of *E. faecalis* growth; (**E**) inhibition zone of *S. aureus* growth; (**F**) inhibition zone of *P. aeruginosa* growth; (**G**) inhibition zone of *L. rhamnosus aeruginosa* growth. Arrows indicate the size of inhibition zones. Note the discoloration of the dressing in panels (**C**–**G**), which occurred as a result of PVP-I release, compared to panel (**A**). Also note the folding of the film in panels (**C**–**G**), which was a result of drying during the incubation period, compared to (**A**).

**Figure 7:**
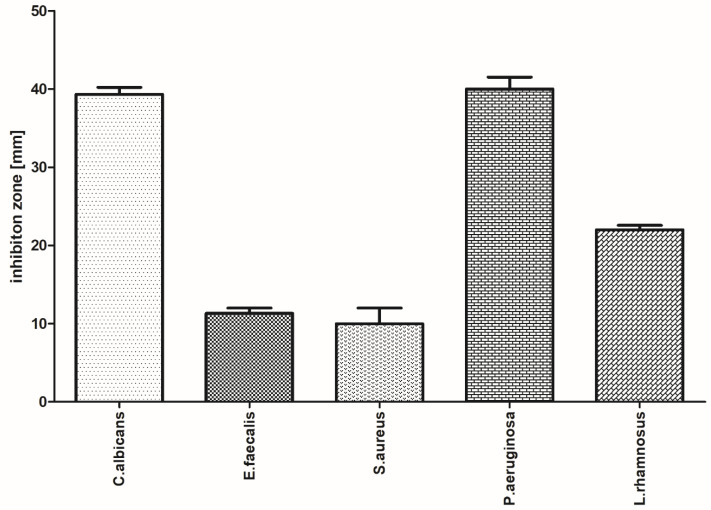
Inhibition zones (mm) of microbial growth generated by PVA/HPMC_B film with incorporated PVP-I (mean ± SD).

The antibacterial activities of the polyvinyl alcohol-hydroxymethyl cellulose film containing PVP-I were tested by the disk-diffusion method against five pathogens, namely, *C. albicans, E. faecalis, P. aeruginosa, L. rhamnosus,* and *S. aureus.* The PVA/HPMC_B film with incorporated PVP-I was shown to possess strong antimicrobial activity against three of the five strains tested. Images highlighting the inhibition of microbial growth and parametric values of zone sizes are presented in Figure 6 and Figure 7, respectively.

The antibacterial activity was calculated based on the formation of a bacterial inhibition zone around the test film, as shown in Figure 7. The PVA-HPMC_B film with incorporated PVP-I shows higher antibacterial action against Gram-negative pathogens (*Candida albicans* and *Pseudomonas aeruginosa*), the functional inhibition of Gram-positive bacteria (*Lactobacillus rhamnosus*), and smaller zones for *Enterococcus faecalis* and *Staphylococcus aureus*.

### 3.4. Assessment of Polymer Material after Subcutaneous Implantation in Rats

**Figure 8:**
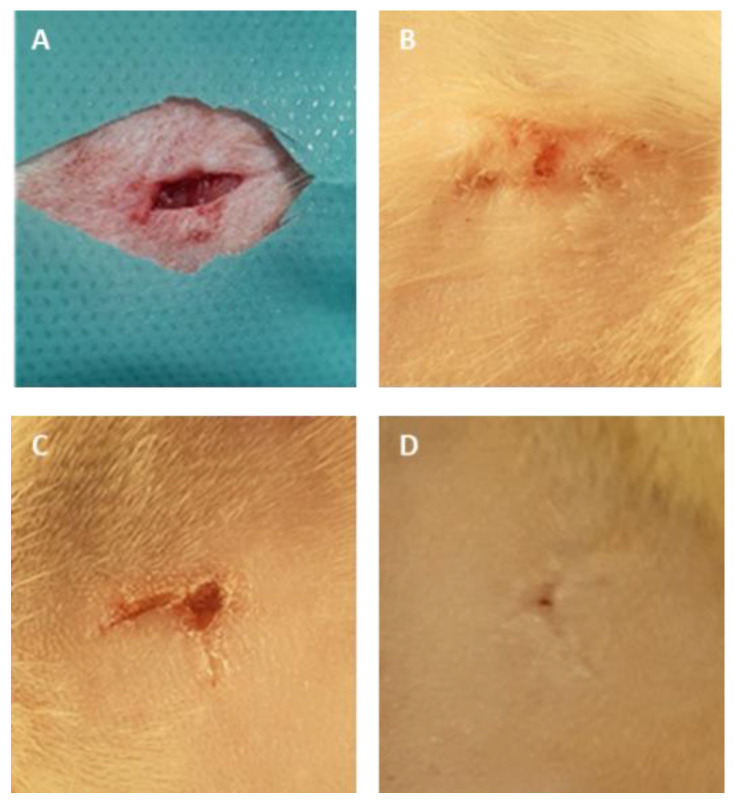
Representative macroscopic images of the skin of rats during and after the implantation procedure. (**A**) The subcutaneous pocket of rat skin created to place films; (**B**) the surface of rat skin 7 days after surgery after the placement of Spongostan; (**C**) placebo film without PVP-I (PVA/HPMC_B/C); (**D**), film with incorporated PVP-I (PVA/HPMC_B/T).

**Figure 9:**
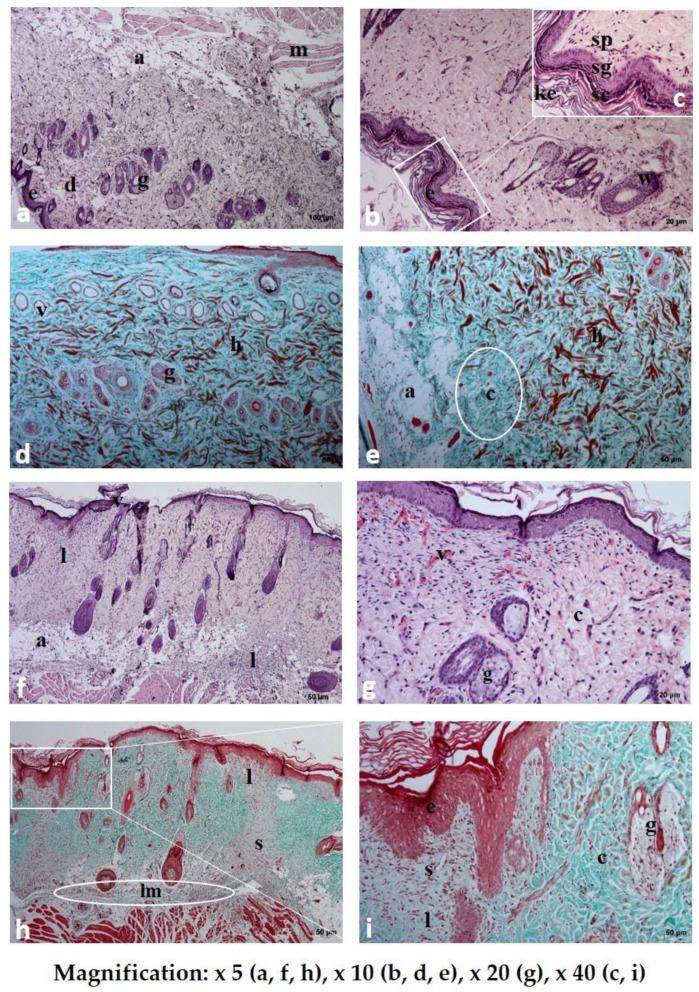
Histological examination of rat skin after the application of Spongostan (**a**–**e**) and placebo film (PVA/HPMC_B/C) (**f**–**i**) stained with hematoxylin and eosin (HE **a**–**c,f,g**) and Masson’s trichrome (**d,e,h,i**) staining: e-epidermis; sc-stratum corneum; sg-stratum granulosum; sp-stratum spinosum; ke-keratin; d-dermis; h-hemorrhagic zones; a-adipose tissue; m-muscular cells; g-sebaceous gland; c-collagen fibers; lm-leukocytes and macrophages; s, ”scar.” In the subcutaneous part of rat skin after the application of placebo film (**h**), a visible layer of inflammatory cells (lm) and a zone with disturbed layers, structurally different from the surrounding, healthy tissues, were observed.

**Figure 10:**
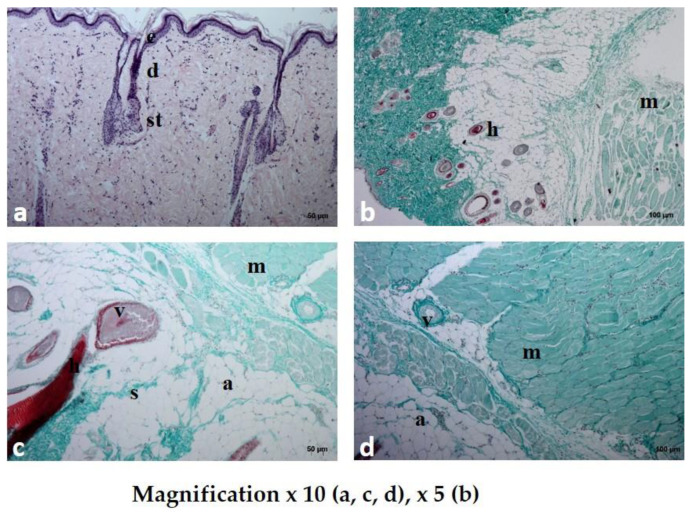
Histological examination of rat skin after the application of PVA/HPMC_B film with incorporated PVP-I (PVA/HPMC_B/T) stained with hematoxylin and eosin (HE) (**a**) and Masson’s trichrome (**b**–**d**) staining. The normal skin with a layered structure without inflammatory cells; e—epidermis; d—dermis; st—subcutaneous tissue; a—adipose tissue; m—muscle cells; h—hair; v—vessel; s—collagen septum.

All 30 rats survived the procedures and were subsequently adopted. The macroscopic images of the skin sections before and 7 days following implantation are shown in Figure 8. In all three groups, the wound healed completely, without redness or inflammation, within a week of surgery. No significant differences in the microscopic analysis were found between the group treated with the placebo film and the group treated with the PVA/HPMC_B film with PVP-I incorporated (Figure 9 and Figure 10). The results of the blood biochemical and morphological analyses are shown in Table 3 and Table 4.

#### 3.4.1. Microscopic Examination of Rat Skin Sections

Dissolution and absorption of the substances present in the investigated materials were observed in all of the studied rat skin sections. The duration of tissue regeneration and the time to return to the baseline differed, depending on the type of formulation used (Figure 9 and Figure 10).

Small hemorrhagic zones and extensive scaling of the stratum corneum were noted in the sections where Spongostan was used. The dye present in the cells of the basal layer formed a uniform, delicate zone, while dye accumulation was only observed in some areas of the stratum corneum. The collagen and elastin fibers in the reticular layer of the dermis were arranged tightly, forming a compact structure without spaces between fibers. There were few inflammatory cells in the skin cross-sections. On the contrary, numerous fibrocytes were present, either dispersed or in the form of vertical “columns” of cells migrating from the adipose layer to the epidermis.

Following dermal placebo film (PVA/HPMC_B/C) administration, a thickened scaling of the epidermis was observed in the specimens. In addition, hemorrhagic zones were visible. The collagen fibers did not form a closely packed layer. The dye did not form a distinct layer, but was dispersed across the entire width of the epidermis. The dermal papillae were enlarged, and skin appendages were present. A “scar” was visible in the form of two vertical areas and one horizontal area, which were structurally and functionally distinct from the surrounding healthy tissue. The scar area lacked skin appendages and contained numerous fibrocytes and tissue macrophages. We observed enlarged adipocytes and numerous fibrocytes scattered within the adipose tissue, as well as several macrophages.

In the samples treated with film with incorporated PVP-I (PVA/HPMC_B/T), the epithelial tissue was structurally unchanged, and there were no hemorrhagic foci. The collagen and elastin fibers formed a loose structure, similar to the healthy, unchanged dermis. Large adipocytes and relatively numerous fibrocytes were present in the adipose tissue. Numerous fibrocytes were present in the muscle tissue. The dermis contained many migrating bands of fibrocytes, small blood vessels, and new skin appendages, indicating an advanced regeneration process compared to the previous two studied section specimens. The presence of capillaries that infiltrated the dissolving film with incorporated PVP-I and began to form a new vascular network indicated stabilization of the wound and a reduction in the risk of infection.

#### 3.4.2. Blood Biochemical and Morphological Analysis

The blood biochemistry was performed using the CatalystOne® (Idexx) machine. All the values were within the reference range (Carpenter J.W., Exotic Animal Formulary. 5 edt, Elsevier 2018). The results are displayed in Table 3. The complete blood count was performed on the ProCyte DX^®^ (Idexx) machine. All the values were within the reference range (Carpenter J.W., Exotic Animal Formulary. 5 edt, Elsevier 2018). The results are presented in Table 4.

## 4. Discussion

In this experimental study, we fabricated films from PVA and hydroxypropyl methylcellulose (PVP-HPMC) or carboxymethylcellulose sodium salt (PVP-CMC). We sought to combine the mechanical and swelling properties of PVA with the flexibility and high water uptake of cellulose derivatives, and assessed the subacute toxicity of the PVP-I carrier composition in a rat model. In earlier studies on base PVA and PVA with CMC, PVA with starch, or PVA with gelatin, PVA with chitosan obtained wound dressings, cryogels, and soft microporous sponges, and reported an excellent mechanical integrity, biocompatibility, and biodegradability, which helped to maintain a moisturized environment near the wounds [35,36,37,38,39]. The dressings we prepared (with PVA-HPMC, as with other combinations described in the literature) are characterized by enhanced properties, i.e., swelling or flexibility relative to pure PVA [40,41]. Povidone-iodine (PVP-I) was selected as the active drug in films, as it displays the broadest spectrum of antimicrobial effects against bacteria, yeasts, molds, other fungi, and certain viruses, and at the same time, microbes lack resistance to the compound [42,43,44,45,46]. Kaeviad et al. developed dental floss impregnated with PVP-I as a drug antimicrobial delivery system against periodontopathogenic bacteria. After floss application, their antimicrobial activity against pathogens, as well as the formation of oral pathogenic biofilms, were observed [47]. Knowledge concerning the impact of various species of microorganisms in the course of aggressive periodontitis is still developing. Besides such bacteria, which are strongly associated with this disease and have been for a long time, such as *Aggregatibacter actinomycetemcomitans* and *Porphyromonas gingivalis* [48], other microbial species are presently recognized as etiological factors of this disease, including *C. albicans, P. aeruginosa* [49], and *E. faecalis* [50]. The role of *S. aureus* in periodontitis is still open for discussion; while some reports indicate the lack of a negative impact of this bacteria [49], others present the contrary [51]. The literature data indicate, in turn, a lack of correlation between the last of the tested species, namely *Lactobacillus*, and periodontitis development [49]. We demonstrated that the PVA-HPMC film with incorporated PVP-I showed a higher level of antibacterial action against Gram-negative pathogens (*Candida albicans* and *Pseudomonas aeruginosa*) in a disk-diffusion in vitro test, and a good inhibition of Gram-positive bacteria (*Lactobacillus rhamnosus*). The implantation of polymer material can be associated with problems such as an acute inflammatory response or sublethal toxicity, and both may last from days to weeks, depending on the type of implanted material. Biodegradable polymer materials are presumed to expel compounds that may induce acute or chronic inflammatory reactions in the host tissue, which may indirectly affect major organs through systemic blood circulation. In this report, to examine the subacute toxicity of selected films, we performed physicochemical and biological tests of PVA-HPMC film containing PVP-I in rats seven days following implantation. Specifically, we analyzed the histological and biochemical parameters after subcutaneous implantation into the skin pocket. The histology, blood biochemical analysis, and blood morphology results indicated that the implanted polyvinyl alcohol-hydroxymethyl cellulose film containing PVP-I may, to a greater extent, have provoked the recruitment of inflammatory mediators to the site of implantation or changed reference values for blood-based biochemical parameters, compared to the commercial dressing Spongostan. Moreover, we confirmed a lack of subacute irritating reactions, as well as a reduction of the inflammation stage when using PVP-I incorporated into the film. Similarly, Chae et al. indicated that PVA/alginate cross-linked hydrogels improved the tissue compatibility by eliciting mild foreign body reactions during acute-phase subcutaneous implantation [52]. The PVA-HPMC films developed offer advantages such as a high biocompatibility, mucoadhesive properties, and a higher resistance of the dressing to blurring after implantation in comparison to semi-solid formulations or commercial dressings.

## 5. Conclusions

In the present work, a polyvinyl alcohol-hydroxypropyl methylcellulose film containing PVP-I 12% (m/m), and glycerol as an excipient, have been successfully developed. The delivered content of iodine and the physicochemical properties of the polyvinyl alcohol-hydroxypropyl methylcellulose film containing PVP-I showed that these formulations were acceptable and suitable for tissue regeneration seven days after subcutaneous implantation in a Wistar rat model. The excipients used in the composition of the developed films resulted in no toxicity in the histopathological analysis compared to the conventional commercially available material Spongostan. In contrast, the inflammation stage was reduced, and the “scar” in the dermis was smaller when the PVA-HPMC film with PVP-I was incorporated. These results indicate that PVA-HPMC film with incorporated PVP-I significantly enhances the tissue healing process and can be used in skin wound treatment. We predict that this device can be administered locally into the periodontal pockets for the safe and efficient management of periodontitis.

## Figures and Tables

**Table 1:** Composition of fabricated films. PVA, polyvinyl alcohol; CMC, carboxymethylcellulose sodium salt; HPMC, hydroxypropyl methylcellulose; GLY, glycerol; PVP-I, povidone-iodine.

Batch Code	PVA	CMC	HPMC	GLY	PVP-I
	[mg per Film]				
PVA/CMC_A	63.2	40.5	-	22.8	12.0
PVA/HPMC_A	63.2	-	40.5	22.8	12.5
PVA/CMC_B	88.5	15.2	-	22.8	12.0
PVA/HPMC_B	88.5	-	15.2	22.8	12.0

**Table 2:** Physical characteristics of the selected polyvinyl alcohol-hydroxypropyl methylcellulose (PVA/HPMC_B) film. Data are presented as the mean ± SE, n = 6.

Physical Characteristics	PVA/HPMC_B
Thickness (mm)	0.46 ± 0.003
Mass (g)	0.1385 ± 0.0043
Disintegration time (min)	110 ± 8.37
pH	6.33 ± 0.32

**Table 3:** The results of the blood biochemical analyses after the application of Spongostan, placebo film (PVA/HPMC_B/C), and film with incorporated PVP-I (PVA/HPMC_B/T).

Group	Altp [U/L]	Alt [[U/L]	Crea [mg/dL]	Bun [mg/dL]	Alb [g/dL]	Glc [mg/dL]	CRP [mg/L]
Spongostan	38.2	93.0	0.596	17.20	4.96	92.6	1.45
PVA/HPMC_B/C	41.4	38.8	0.590	15.68	5.01	85.7	1.69
PVA/HPMC_B/T	43.0	38.4	0.614	16.08	4.93	85.6	1.59

Altp, aspartate aminotransferase; Alt, alanine aminotransferase; Crea, creatinine; Bun, blood urea nitrogen; Alb, albumin; Glc, blood glucose level; CRP, C-reactive protein.

**Table 4:** The results of the blood morphological analyses after the application of Spongostan, placebo film (PVA/HPMC_B/C), and film incorporated with PVP-I (PVA/HPMC_B/T).

Group	RBC (10^6^/μL)	WBC (10^3^/μL)	HGB (g/dL)	MPV (%)
Spongostan	8.06	10.012	15.07	40.11
PVA/HPMC_B/C	8.15	10.108	15.22	40.20
PVA/HPMC_B/T	8.45	10.750	14.83	40.41

WBC, white blood cell; RBC, red blood cell; HGB, hemoglobin; MPV, mean platelet volume.
